# Hedgehog Pathway Activation Alters Ciliary Signaling in Primary Hypothalamic Cultures

**DOI:** 10.3389/fncel.2019.00266

**Published:** 2019-06-12

**Authors:** Ruchi Bansal, Staci E. Engle, Patrick J. Antonellis, Logan S. Whitehouse, Anthony J. Baucum, Theodore R. Cummins, Jeremy F. Reiter, Nicolas F. Berbari

**Affiliations:** ^1^Department of Biology, Indiana University – Purdue University Indianapolis, Indianapolis, IN, United States; ^2^Stark Neurosciences Research Institute, Indianapolis, IN, United States; ^3^Department of Biochemistry and Biophysics, Cardiovascular Research Institute, University of California, San Francisco, San Francisco, CA, United States; ^4^Center for Diabetes and Metabolic Disorders Research, Indiana University School of Medicine, Indianapolis, IN, United States

**Keywords:** cilia, primary neuronal cultures, hypothalamus, leptin, melanin concentrating hormone receptor 1, hedgehog signaling, smoothened, SAG

## Abstract

Primary cilia dysfunction has been associated with hyperphagia and obesity in both ciliopathy patients and mouse models of cilia perturbation. Neurons throughout the brain possess these solitary cellular appendages, including in the feeding centers of the hypothalamus. Several cell biology questions associated with primary neuronal cilia signaling are challenging to address *in vivo*. Here we utilize primary hypothalamic neuronal cultures to study ciliary signaling in relevant cell types. Importantly, these cultures contain neuronal populations critical for appetite and satiety such as pro-opiomelanocortin (POMC) and agouti related peptide (AgRP) expressing neurons and are thus useful for studying signaling involved in feeding behavior. Correspondingly, these cultured neurons also display electrophysiological activity and respond to both local and peripheral signals that act on the hypothalamus to influence feeding behaviors, such as leptin and melanin concentrating hormone (MCH). Interestingly, we found that cilia mediated hedgehog signaling, generally associated with developmental processes, can influence ciliary GPCR signaling (Mchr1) in terminally differentiated neurons. Specifically, pharmacological activation of the hedgehog-signaling pathway using the smoothened agonist, SAG, attenuated the ability of neurons to respond to ligands (MCH) of ciliary GPCRs. Understanding how the hedgehog pathway influences cilia GPCR signaling in terminally differentiated neurons could reveal the molecular mechanisms associated with clinical features of ciliopathies, such as hyperphagia-associated obesity.

## Introduction

The hypothalamus is a complex brain region consisting of several nuclei that regulate food and water intake, circadian rhythm, sexual behavior, and body temperature. Two hypothalamic nuclei involved in feeding behavior, the arcuate nucleus (ARC) and the paraventricular nucleus (PVN), contain several neuronal populations that respond to neuropeptides, and hormones produced both centrally and peripherally (Schwartz et al., 2000; Andermann and Lowell, 2017). Primary neuronal cultures are a valuable tool to help better understand the cell biology of how hypothalamic neurons respond to these stimuli and ultimately regulate feeding behaviors. For example, the mechanisms underlying genetic disorders associated with hyperphagia and obesity, such as Bardet-Biedl syndrome (BBS) and Alström syndrome (ALMS) remain unclear. Both BBS and ALMS are classified as ciliopathies, as primary cilia dysfunction is thought to be the cellular etiology of the disorders (Vaisse et al., 2017). Primary cilia are small microtubule based cellular appendages critical to coordinating diverse signaling pathways on many cell types in nearly every organ system, including neurons of the CNS (Singla and Reiter, 2006; Berbari et al., 2009). Hypothalamic neuronal cilia appear critical to normal feeding behavior. Both conditional mouse models of BBS and cilia loss become hyperphagic and obese (Mykytyn et al., 2004; Davenport et al., 2007; Berbari et al., 2013; Guo et al., 2016). Intriguingly, congenital loss of primary cilia from POMC neurons also results in hyperphagia and obesity (Davenport et al., 2007). How primary cilia on these neurons regulate food intake is poorly understood, but several G-protein coupled receptors (GPCRs) have been found to preferentially localize to primary cilia, including melanin concentrating hormone receptor 1 (Mchr1) and melanocortin receptor 4 (Mc4r), both of which are expressed in the hypothalamus along with their ligands, MCH and alpha-melanocyte stimulating hormone (α-MSH), respectively, and have established roles in feeding behavior and obesity (Desy and Pelletier, 1978; Bittencourt et al., 1992; Berbari et al., 2008a; Siljee et al., 2018).

*In vitro* model systems of primary cilia have helped elucidate the sensory and regulatory role these organelles play in different cell types (Praetorius and Spring, 2001; Rohatgi et al., 2007). Primary cultures of the hippocampus have been useful in characterizing primary cilia *in vitro* (Berbari et al., 2007; Yao et al., 2015). However, primary hypothalamic cultures have not been as widely used. Here we aim to establish an *in vitro* system using cultured hypothalamic neurons to understand new aspects of cilia signaling which may impact feeding behavior. We compare primary cultures obtained from the hippocampus to the hypothalamus and show that hypothalamic cultures express significantly higher levels of region-specific genes, such as POMC and AgRP. Moreover, we show primary hypothalamic cultures possess primary cilia and are responsive to both peripheral (leptin) and central (MCH) neuropeptides. For the first time, we demonstrate that the cilia mediated hedgehog pathway agonist, SAG, attenuates normal electrophysiological responses to a ciliary GPCR ligand in terminally differentiated neurons. These data suggest an integration between the hedgehog pathway and ciliary GPCR signaling in terminally differentiated neurons.

## Results

### Hypothalamus Derived Cultures Contain Populations of Region-Specific Neurons and Glia

**FIGURE 1:**
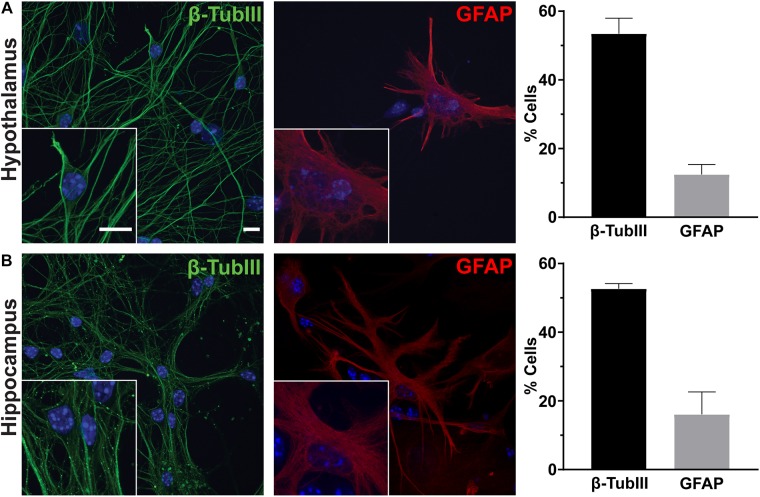
Hypothalamic and hippocampal culture content. Hypothalamic **(A)** and hippocampal **(B)** cultures from perinatal mice contain neurons [β-tubulin III (β-TubIII), green] and glia [glial fibrillary acidic protein (GFAP), red] after 7 days in culture. The percentage of cells positive for the associated stain divided by the total number of cells counted per hypothalamus or hippocampus is graphed as mean ± SEM. Hoechst stained nuclei are blue. Scale bars are 10 μm. Data collected from 3 hypothalamic and hippocampal samples from 3 experimental days. β-tubulin III positive cells/total hypothalamic nuclei: 336/628, GFAP positive cells/total hypothalamic nuclei: 126/647, β-tubulin III positive cells/total hippocampal nuclei: 323/613, and GFAP positive cells/total hippocampal nuclei: 108/617.

**FIGURE 2:**
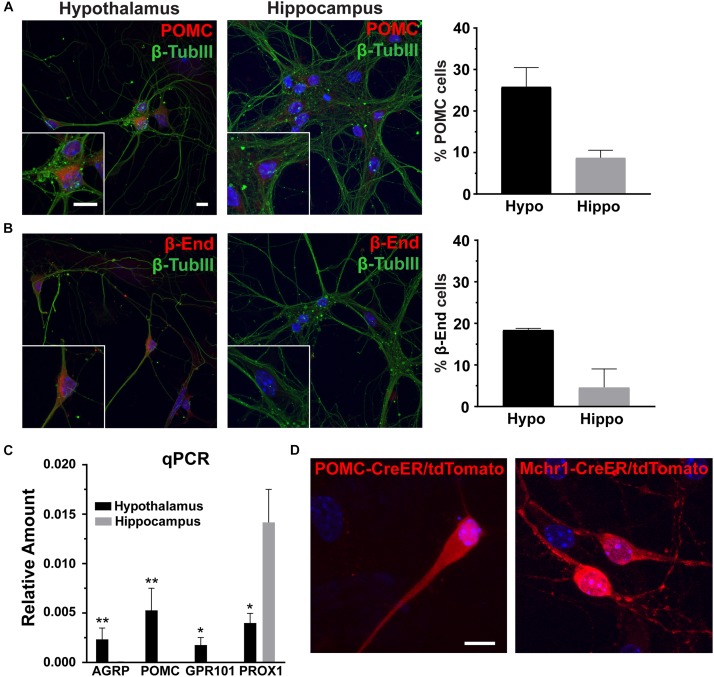
Hypothalamic characteristics *in vitro.* **(A)** Proopiomelanocortin (POMC, red) and **(B)** β-endorphin (β-End, red) staining in hippocampal and hypothalamic cultures co-stained for β-tubulin III (β-TubIII, green). Scale bars are 10 μm. Percentage of cells positive for POMC or β-endorphin divided by the total number of cells counted in the hypothalamus and hippocampus graphed as mean ±SEM. Data collected from 3 hypothalamic and hippocampal samples from 3 experimental days. POMC positive cells/total hypothalamic nuclei: 166/642, β-endorphin positive cells/total hypothalamic nuclei: 203/657, POMC positive cells/total hippocampal nuclei: 57/653, and β-endorphin positive cells/total hippocampal nuclei: 69/678. **(C)** Quantitative PCR (qPCR) analysis on total RNA isolated from day 7 hypothalamic and hippocampal cultures. Gene expression of agouti related peptide (AgRP), POMC, G protein-coupled receptor 101 (GPR101), and Prospero homeobox protein 1 (PROX1) relative to actin is graphed as mean ± SEM. ^*^P < 0.05 and ^∗∗^*P* < 0.01 using a Students *t*-test. Data collected from 7 hypothalamic and hippocampal samples from 3 experimental days. **(D)** Neurons cultured from mice with hypothalamic inducible cre alleles (POMC-CreER and Mchr1-CreER) show the presence of reporter (tdTomato) expression *in vitro* upon 4-OH tamoxifen treatment. In all images Hoechst stained nuclei are blue and scale bars are 10 μm.

To determine the utility of hypothalamic neuronal cultures, we compared hypothalamic cultures to hippocampal cultures from the same animals (Figure 1). Similar percentages of both neurons and glia were present, as observed by β-tubulin III and GFAP staining (Figures 1A,B). To determine if hypothalamic cultures expressed region specific peptides *in vitro*, we stained for both POMC and β-endorphin and found more cells expressing these neuropeptides when compared to hippocampal-derived controls (Figures 2A,B). Similar results were observed using quantitative PCR for hypothalamic gene expression (Figure 2C). To further confirm that neurons from multiple hypothalamic regions were present *in vitro*, we utilized Cre recombinase inducible alleles with a tdTomato fluorescent reporter for both POMC and Mchr1 expressing neurons which are found in the ARC and PVN *in vivo* and reporter positive cells were observed *in vitro* (Berglund et al., 2013; Engle et al., 2018; Figure 2D). Together, these results suggest that similar to cultured hippocampal primary neurons, hypothalamus derived neurons display expression patterns like those observed *in vivo*.

### Hypothalamic Neurons in Culture Respond to Leptin

**FIGURE 3:**
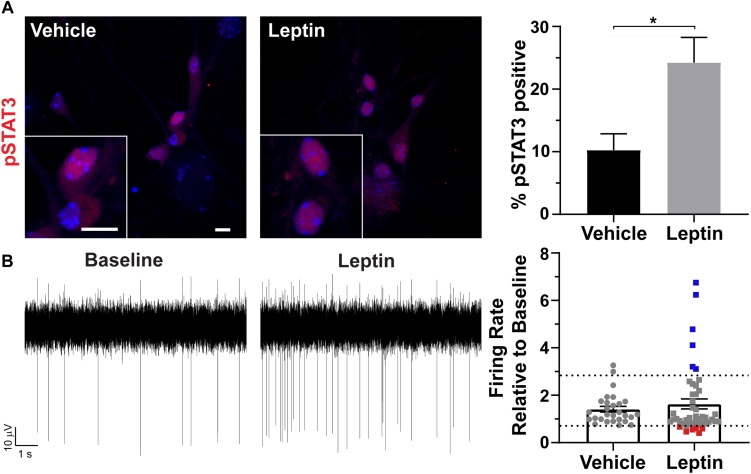
Hypothalamic specific responses to leptin *in vitro.* **(A)** Phosphorylation and nuclear localization of STAT3 (pSTAT3, red) following 30 min leptin (10 nM) treatment. Hoechst stained nuclei are blue and scale bars are 10 μm. Percent pSTAT3 positive cells in vehicle and leptin treated cultures divided by total number of cells counted is graphed as mean ± SEM. ^*^P < 0.05 using a Student’s *t*-test. Data collected from 3 hypothalamic samples from 3 experimental days. pSTAT3 positive cells/total vehicle treated hypothalamic nuclei 34/333, pSTAT3 positive cells/total leptin treated hypothalamic nuclei 86/354. **(B)** Representative traces from one electrode showing the baseline activity and activity after 10 nM leptin addition. The change in firing rate relative to baseline is graphed as mean ± SEM for vehicle and leptin addition. Each point represents an active electrode. Dashed line indicates two time above and below the average of the vehicle bar. Leptin results in some electrodes displaying decreases (red squares), increases (blue squares), or no change (gray squares) in neuronal activity relative to baseline. Data collected from 4 hypothalamic samples from 3 experimental days.

To determine if cultured neurons are responsive to neuropeptides, we treated them with leptin and assessed phosphorylation, and nuclear localization of STAT3 (pSTAT3) (Banks et al., 2000; Pissios et al., 2003). pSTAT3 was significantly increased after leptin treatment (Figure 3A). To further assess if these neurons were also electrophysiologically active and capable of responding with changes in firing similar to those observed *in vivo*, we isolated the neurons onto multi-electrode arrays (MEAs) and recorded extracellular potentials (Spira and Hai, 2013). In patch clamp recording experiments, AgRP neurons decrease activity, and POMC neurons increase activity in response to leptin (Cowley et al., 2001). Assessment of our MEA data revealed greater variation in the change in firing rate in response to leptin addition compared to vehicle addition (Figure 3B). We observed predictable responses to leptin. We considered any activity changes twice that of vehicle to be an increase (blue points on Figure 3B graph) while changes half of the vehicle were considered to be a decrease (red points on Figure 3B graph). Electrode responses between those groups (gray points on Figure 3B) either did not change with leptin or are the result of changes from individual cells canceling each other out in the overall extracellular field potential recorded by that electrode. These results demonstrate that cultured hypothalamic neurons are capable of responding to classical neuropeptides, such as leptin, involved in feeding behavior and energy homeostasis.

### Hypothalamic Neurons Possess Cilia in Culture

**FIGURE 4:**
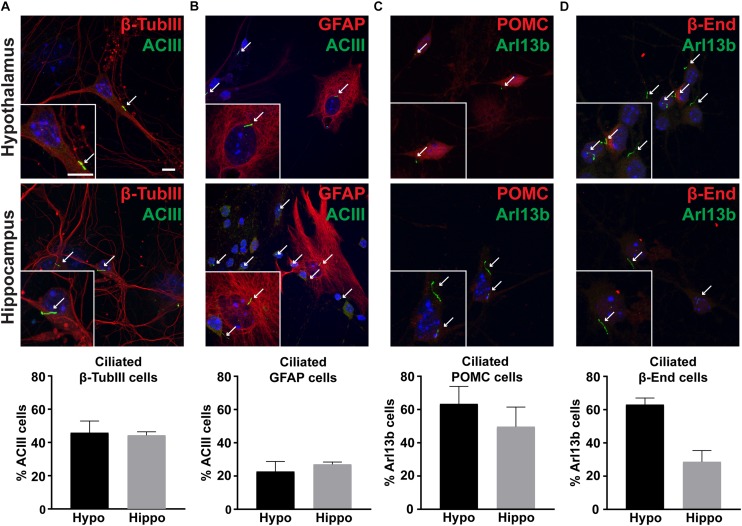
Cultures possess primary cilia. **(A)** β-tubulin III (β-TubIII, red) stained neurons with adenylyl cyclase III (ACIII, green) positive cilia. **(B)** Glial fibrillary acidic protein (GFAP, red) stained astrocytes with adenylyl cyclase III (ACIII, green) positive cilia. **(C)** Proopiomelanocortin (POMC, red) expressing, and **(D)** β-endorphin (β-End, red) expressing neurons with ADP Ribosylation Factor Like GTPase 13B (Arl13b, green) positive cilia in both hypothalamic and hippocampal derived neurons. Hoechst stained nuclei are blue. Arrows indicate cilia. Scale bars are 10 μm. Percentage of cilia marker positive cells divided by the total number of associated cell type assessed per hypothalamus or hippocampus is graphed as mean ± SEM. Data collected from 3 hypothalamic and hippocampal samples from 3 experimental days. β-tubulin III cells with ACIII positive cilia/total hypothalamic β-tubulin III positive cells: 152/336, β-tubulin III cells with ACIII positive cilia/total hippocampal β-tubulin III positive cells: 160/323, GFAP cells with ACIII positive cilia/total hypothalamic GFAP positive cells: 32/126, GFAP cells with ACIII positive cilia/total hippocampal GFAP positive cells: 42/108, POMC cells with Arl13b positive cilia/total hypothalamic POMC positive cells: 100/166, POMC cells with Arl13b positive cilia/total hippocampal POMC positive cells: 28/57, β-endorphin cells with Arl13b positive cilia/total hypothalamic β-endorphin positive cells: 124/203, and β-endorphin cells with Arl13b positive cilia/total hippocampal β-endorphin positive cells: 37/69.

To determine if hypothalamic neurons possess primary cilia *in vitro*, we stained hypothalamic and hippocampal cells with cilia specific markers such as adenylyl cyclase III (ACIII) or ADP Ribosylation Factor Like GTPase 13B (Arl13b) (Bishop et al., 2007; Caspary et al., 2007). Similar to other neuronal culture systems, we observed nearly half of β-Tubulin III positive neurons possessed cilia (Figure 4A; Berbari et al., 2007). In contrast, only 25% of GFAP positive astrocytes possessed ACIII positive cilia (Figure 4B). To determine if POMC neurons *in vitro* possess primary cilia, we also labeled these cultures with POMC or β-endorphin antibodies. We found that nearly 60% of hypothalamic neurons expressing either POMC or β-endorphin also possess Arl13b positive cilia (Figures 4C,D). These data confirm that neurons in hypothalamic cultures possess primary cilia, similar to those derived from the hippocampus and nucleus accumbens (Berbari et al., 2007; Yao et al., 2015).

### Cilia Mediated Hedgehog Pathway Agonism Impacts Cilia GPCR Mediated Neuronal Activity

**FIGURE 5:**
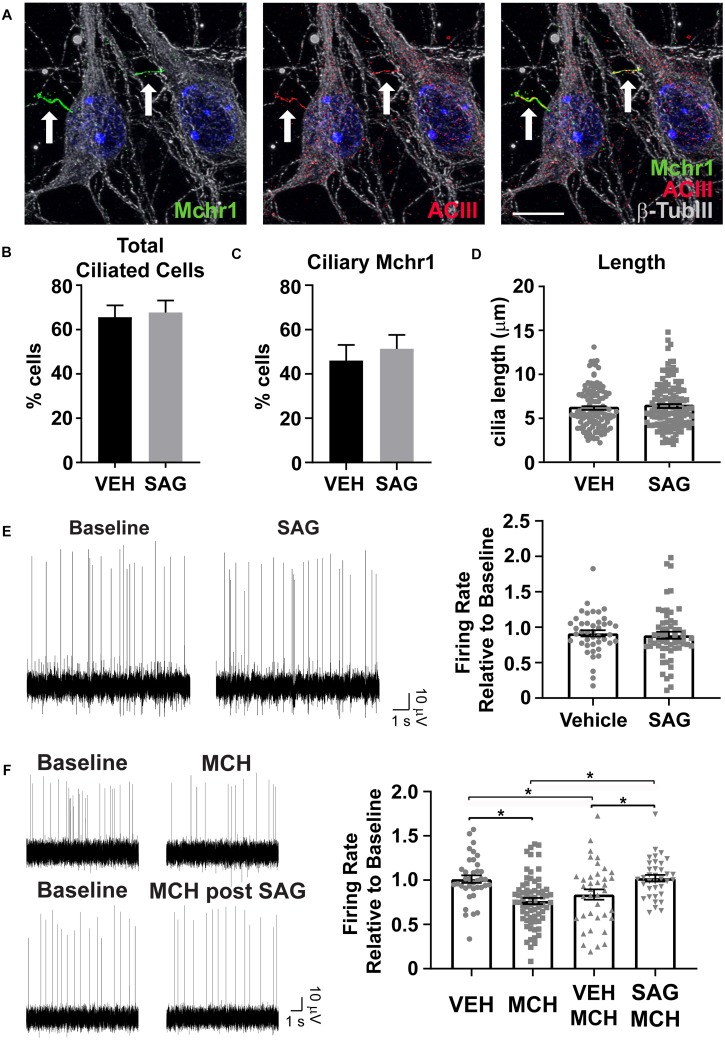
Hedgehog pathway agonism attenuates neuronal response to MCH. **(A)** Melanin concentrating hormone receptor 1 (Mchr1, green) localizes to primary cilia (ACIII, red) of cultured hypothalamic neurons labeled with β-tubulin III (β-TubIII, gray). Hoechst stained nuclei blue. Scale bar 10 μm. Arrows indicate cilia. **(B)** Cilia counts in hypothalamic cultures following 24 h SAG treatment. Percent cilia positive cells in vehicle and SAG treated cultures is graphed as mean ± SEM. Vehicle treated hypothalamic cultures: 121 ACIII positive cilia/189 total cells. SAG treated hypothalamic cultures: 127 ACIII positive cilia/189 total cells. **(C)** Mchr1 positive cilia following SAG treatment. Percent Mchr1 positive cilia in vehicle and SAG treated cultures are graphed as mean ± SEM. Vehicle treated hypothalamic cultures: 83 Mchr1 positive cilia/121 ACIII positive cilia. SAG treated hypothalamic cultures: 96 Mchr1 positive cilia/127 ACIII positive cilia. **(D)** Cilia length measurements. Graphed as mean ± SEM. Outliers were identified and removed using ROUT with Q = 1%. 121 cilia were measured following 24 h vehicle treatment and 127 cilia were measured following 24 h SAG treatment. **(E)** Representative traces from one electrode showing baseline activity and activity after addition 400 nM SAG. The change in firing relative to baseline is graphed as mean ± SEM for vehicle and SAG addition. **(F)** Representative traces from one electrode showing baseline activity and activity after addition of 1 μM MCH or MCH following 24 h pretreatment with 400 nM SAG. The change in firing relative to baseline is graphed as mean ± SEM for vehicle and peptide addition. Each point represents an active electrode. ^*^P < 0.05 using an Ordinary one-way ANOVA with Tukey’s multiple comparisons test. Outliers were identified and removed using ROUT with Q = 1%. All data collected from 3 to 6 hypothalamic samples from 3 to 5 experimental days.

Several GPCRs preferentially localize to primary cilia of neurons throughout the brain, including Mchr1 (Berbari et al., 2008b; Hamamoto et al., 2016; Tomoshige et al., 2017). We observe Mchr1 colocalizing with the cilia marker ACIII (Figure 5A). In olfactory neurons changes in cilia mediated hedgehog signaling altered GPCR localization to cilia (Maurya et al., 2017). To see if activation of the hedgehog pathway affected the number of Mchr1 positive cilia in hypothalamic neurons, we treated cultures with 400 nM smoothened agonist (SAG) for 24 h and immunolabelled with Mchr1 and ACIII. We did not see any change in the percentage of ciliated cells after treatment with SAG nor did we see any difference in the number of Mchr1 positive cilia (Figures 5B,C). We next assessed if SAG treatment could impact cilia length or electrophysiological activity. Published data suggests that alterations in neuronal cilia length can influence their ability to signal (Besschetnova et al., 2010; Tomoshige et al., 2017). However, we did not observe cilia length changes with SAG treatment (Figure 5D). Furthermore, SAG treatment alone did not impact the firing rate of hypothalamic cultures (Figure 5E). Interestingly we saw activation of the hedgehog pathway can influence responses to MCH. MCH (1 μM) significantly decreases the firing rate of hypothalamic cultures (Figure 5F), similar to observations made in slice recordings (Gao and van den Pol, 2001). However, MCH elicits no change when cultures were pretreated with SAG (Figure 5F). These results suggest that there may be functional integration between ciliary GPCR signaling and the hedgehog pathway. Future studies will investigate the physiological relevance of persistent hedgehog pathway expression in terminally differentiated hypothalamic neurons as well as the ability for hedgehog signaling to influence other ciliary GPCRs.

## Discussion

The ability to culture primary rodent neurons that establish and maintain their hypothalamic specific identity, responsiveness and activity *in vitro* has been limited. Here we applied improved culturing techniques and utilized a media developed specifically to enhance neuronal activity *in vitro* (Bardy et al., 2015). We confirmed that hypothalamic derived neurons expressed hypothalamic genes such as β-endorphin and that they responded to central (MCH) and peripheral (leptin) signals with changes in spontaneous firing as occurs in slice recordings (Cowley et al., 2001; Gao and van den Pol, 2001; Davidowa et al., 2002, 2003). The ability to culture hypothalamic derived primary neurons that establish and maintain their identity and responsiveness can be utilized for rapidly assessing genetic perturbation and pharmacological responses. The use of MEAs offers the ability to rapidly analyze network effects which can then be followed up using more time consuming and expensive traditional electrophysiological approaches or leading-edge DREADD and optogenetic approaches *in vivo* (Bernstein and Boyden, 2011; Spira and Hai, 2013; Roth, 2016).

We sought to use these neurons to learn how primary cilia mediated signaling pathways may potentially interact. Dysfunction of primary cilia leads to hyperphagia and obesity in patients with genetic disorders such as BBS and ALMS (Vaisse et al., 2017). Nearly half of neurons and a quarter of glial cells possess ACIII positive primary cilia, similar to what we see in our hippocampal cultures and has been previously reported in cultured hippocampal neurons (Berbari et al., 2007). It is likely that even more cultured cells possess primary cilia, as ACIII is not a universal neuronal cilia marker (Antal et al., 2017). Not only do our cultured hypothalamic neurons possess primary cilia but we also see Mchr1 localize to the cilia in agreement with previous studies (Berbari et al., 2008a, b). The MCH pathway plays a recognized role in feeding behavior (Qu et al., 1996; Chen et al., 2002); however, the role of ciliary localization of Mchr1 is poorly understood. Primary hypothalamic cultures will help to address cell biology questions regarding ciliary vs. non-ciliary Mchr1 signaling in relevant cell types.

One well-studied pathway that is coordinated by primary cilia is the hedgehog pathway (Huangfu et al., 2003; Huangfu and Anderson, 2005; Goetz and Anderson, 2010). This pathway is crucial for embryonic development (Szabo et al., 2009) but there may be important roles for the pathway in postmitotic neurons as well. For example, hedgehog pathway components are found in adult hippocampal cells and treatment with hedgehog ligand can increase the excitability of cultured hippocampal neurons (Mitchell et al., 2012). Another study showed that the agonist SAG decreased the firing rate of cultured cortical neurons (Ugbode et al., 2017), further supporting that the hedgehog pathway can influence neuronal activity. In addition to the idea that the hedgehog pathway may alter neuronal firing, there are other studies that led us to hypothesize that it perhaps can influence GPCR signaling in postmitotic cells. For example, GPR161, is critical for proper hedgehog signaling in development (Mukhopadhyay et al., 2013; Pusapati et al., 2018). In addition, conditional deletion of the smoothened gene from olfactory sensory neurons leads to a decrease of odorant GPCR localization to olfactory cilia (Maurya et al., 2017). We are interested in understanding if similar connections exist between hedgehog signaling and cilia GPCRs in neurons. Here we report for the first time that hedgehog pathway agonism can attenuate neuronal responses to MCH. From our data it appears that a subpopulation of cells in hypothalamic cultures respond to MCH with a decrease in firing rate and that SAG treatment can block that response. Mchr1 is expressed on different cell types in different nuclei of the hypothalamus, some of which include AgRP and POMC neurons within the ARC and several types of neurons within the PVN (Chee et al., 2013). It is unclear which cell type or types are impacted by MCH and SAG treatment and this will be the focus of future studies.

Activation of the hedgehog pathway involves ligand dependent dynamic localization of both the receptor, patched, and mediator smoothened, out of and into primary cilia (Corbit et al., 2005; Rohatgi et al., 2007). We originally predicted that Mchr1 can also dynamically localize and that SAG treatment may cause it to move out of the cilia, resulting in a dampened response to MCH following pretreatment with hedgehog agonists. If this were happening, we would have expected to see fewer cilia containing Mchr1 in the SAG treated cultures. However, we did not observe changes in total cilia number, Mchr1 labeled cilia, or cilia length. Therefore, we do not think that dynamic localization of Mchr1 out of the cilia is responsible for the lack of electrophysiological response to ligand following SAG treatment. There are other possibilities for how SAG could be impeding Mchr1 signaling. Proper hedgehog signaling relies on post-translational modifications (Hsia et al., 2015; Arensdorf et al., 2016). It would be interesting if activation of the hedgehog pathway also resulted in post-translational modifications to ciliary GPCRs, like Mchr1, and thus change its downstream signaling. In addition, several GPCRs can biochemically interact including those within the cilium (Green et al., 2012; Gomes et al., 2016). Perhaps when smoothened, itself a seven transmembrane protein, is activated it moves to the cilia and heterodimerizes with Mchr1 causing a change in signaling capabilities. However, our staining results for smoothened upon SAG treatment suggest that there are relatively few smoothened positive cilia (data not shown). It is possible that antibody staining for either Mchr1 or smoothened in the cilia is technically limiting due to changes in epitopes of interacting GPCRs. Alternatively, it is also possible that SAG acts on non-Mchr1 expressing cells that then inhibit the firing of Mchr1 neurons. Future studies will involve exploring the mechanism of how hedgehog pathway components may interact or influence not only Mchr1 signaling, but also signaling of other GPCRs known to preferentially localize to primary cilia such as Drd1, Kiss1r, 5-Htr6, Npy5r, Mc4r, and Sstr3 (Hamon et al., 1999; Handel et al., 1999; Brailov et al., 2000; Schulz et al., 2000; Marley and von Zastrow, 2010; Domire et al., 2011; Loktev and Jackson, 2013; Koemeter-Cox et al., 2014; Siljee et al., 2018). Ultimately, experiments addressing the *in vivo* relevance of any observed interactions are needed.

## Materials and Methods

### Animals

Wildtype primary cultures were obtained from C57BL/6J mice from Jackson Laboratories. Cre lines Pomc-CreER (generously provided by Joel K. Elmquist and Chen Liu) and Mchr1-CreER have both been previously described (Berglund et al., 2013; Engle et al., 2018). All procedures and approaches were approved by the Institutional Animal Care and Use Committee at Indiana University-Purdue University School of Science, Indianapolis.

### Primary Neuronal Culture

Primary neuronal cultures were established as previously reported (Berbari et al., 2007, 2008b). Briefly, on the day of birth, mice were decapitated and the hypothalamus and hippocampus were placed in 0.2 mg/mL bovine serum albumin (Sigma-Aldrich, St. Louis, MO, United States) in Leibovitz’s L-15 media (L15/BSA) (Gibco, Gaithersburgh, MD, United States). The tissue was then transferred to L15/BSA with 0.375 mg/mL papain (Sigma-Aldrich) under 95% O_2_/5% CO_2_ gas for 10 min at 37°C. This was followed by two washes in Earle’s Minimal Essential Medium (Gibco) containing 5% fetal bovine serum (Gibco), 5% horse serum (Gibco), 400 μM glutamax (Gibco), 16.65 mM glucose (Sigma-Aldrich), and 2.5 μl/ml insulin/transferrin/selenite (Sigma-Aldrich), collectively called M5-5 media. The cells were mechanically dissociated by trituration through a series of three flame polished Pasteur pipettes with each subsequent pipette having a smaller bore size. The cells were then spun down at 120 × *g* for 5 min at room temperature, pellets were resuspended in M5-5 media (2 mL/hypothalamus, 3 mL/hippocampus), and plated on poly-L-Lysine (Sigma-Aldrich) coated coverslips in 24 well plates. Hypothalamic cells were plated on two coverslips per animal at a starting density of 300,000 cells/well while hippocampal cells were distributed on three coverslips per animal at a starting density of 400,000 cells/well. Two days later, half of M5-5 media was removed and replaced with 1 mL of Neurobasal media (Gibco) supplemented with 2% B27 supplement (Gibco), 2.5 μl/ml insulin/transferrin/selenite, 500 μM glutamax, and 1 μl/ml gentamycin (Gibco) for immunostaining approaches. The media was supplemented with 10 μM cytosine arabinofuranoside (ARA-C, Sigma-Aldrich) to prevent cell proliferation. Cells were incubated at 37°C and 5% CO_2_ for 7–10 days for immunofluorescence studies or 10–14 days for electrophysiological studies.

### CreER Induction

To induce CreER *in vitro*, neurons were treated with 0.5 μM 4-hydroxytamoxifen (Sigma Aldrich) (Danielian et al., 1998) at 5 days in culture and fixed on day 7 for immunofluorescence as described below.

### Immunofluorescence

Immunofluorescence was performed as previously reported (Berbari et al., 2008a). Briefly, after 7–10 days in culture, cells were fixed for 10 min at room temperature with a solution of either 4% paraformaldehyde (PFA;Affymetrix/USB, Cleveland, OH, United States)/10% sucrose (Dot Scientific Inc, Burton, MI, United States) or when staining with the Mchr1 antibody 4% PFA/Histochoice (Sigma-Aldrich). Fixation was followed by incubation in 100% methanol (Thermo Fisher Scientific) for 15 min at −20°C. The cells were then permeabilized and blocked with 0.1% Triton X-100 (Thermo Fisher Scientific) in PBS for 6 min and blocked in PBS containing 1% BSA, 0.1% Triton X-100, 2% donkey serum, and 0.02% sodium azide (Dot Scientific) for at least half an hour. Cells were then incubated in primary antibodies in blocking solution overnight at 4°C. Following 3 five-minute washes in PBS, the cells were incubated with secondary antibodies in blocking solution for 1.5 h at room temperature. Nuclei were stained with Hoechst 33342 (Thermo Fisher Scientific, Waltham, MA, United States) and cells were mounted on slides using 1,4-diazabicyclo[2.2.2]octane (DABCO) and sealed with nail polish or ProLong Diamond (Invitrogen, Carlsbad, CA, United States). Primary cilia were stained with either Arl13b (mouse, 1:300; UC Davis/NIH NeuroMab Facility, Davis, CA, United States 75-287), ACIII (rabbit, 1:500; Santa Cruz Biotechnology, Dallas, TX, United States sc-588), ACIII (rabbit, 1:5000; EnCor Biotechnology, Gainsville, FL, United States RPCA_ACIII), or Mchr1 (goat, 1:250; Santa Cruz Biotechnology sc-5534). Neurons were detected using β-endorphin (rabbit, 1:250; Phoenix Pharmaceuticals, Burlingame, CA, United States H-022-33), POMC (rabbit, 1:250; Phoenix Pharmaceuticals H-029-30), or β-tubulin III (mouse, 1:1000; Sigma Life Sciences, St. Louis, MO, United States T8660) antibodies. Glial cells were stained with GFAP (mouse, 1:1000; Sigma Life Sciences G3893) antibody. Induction of phosphorylation of STAT3 was identified by staining cells with pSTAT3 (mouse, 1:200; Thermo Fisher Scientific MA5-15193). The secondary antibodies used were donkey anti-mouse Alexa 488 (Thermo Fisher Scientific, Waltham, MA, United States A21202), donkey anti-rabbit Alexa 546 (Thermo Fisher Scientific A10040), donkey anti-rabbit Alexa 488 (Thermo Fisher Scientific A21206), donkey anti-mouse Alexa 546 (Thermo Fisher Scientific A10036), donkey anti-goat Alexa 546 (Thermo Fisher Scientific A11056), and donkey anti-mouse Alexa 647 (Thermo Fisher Scientific A31571) at 1:1000 dilution. Control experiments included secondary antibody only.

### Imaging

Cultured neurons were imaged with a Leica SP8 confocal microscope in resonant scanning mode using a 63× NA 1.4 objective. For all images collected, contrast and intensity were adjusted using ImageJ. For counting cells, samples were imaged with a Nikon 90i epifluorescent microscope equipped with a Hamamatsu Orca Flash4-LT CMOS camera, SOLA-SE II LED illuminator.

### Cell and Cilia Counts

Fields were randomly selected from hypothalamic and hippocampal cultures. The total number of positively stained cells were counted and the results were tabulated as percentage by dividing by the total number of cells selected. ImageJ was used to measure the intensity of pSTAT3 staining in the nucleus of vehicle and leptin treated cultures. Any nuclei with a value twice as high as average background intensity was counted as pSTAT3 positive.

### Cilia Length Measurements

Z-stack images of cilia were collected with a Leica SP8 confocal microscope in resonant scanning mode using a 63× NA 1.4 objective. Images were collected in 0.2 μm thick slices and pixel size for each stack was kept at 45 nm × 45 nm. The images were then deconvolved using Lightning Deconvolution module in Leica Application Software (LAS X). Cilia lengths were then measured in a three-dimensional space using 3D measurement module in LAS X by manually tracing along the lengths of the cilia.

### RT-qPCR

RNA was isolated using the RNeasy Micro Kit (Qiagen, Venlo, Netherlands) and then reverse transcribed into cDNA using the Quantitect Reverse Transcription Kit (Qiagen). Reactions were performed on an Applied Biosystems QuantStudio 7 RealTime PCR System (Applied Biosystems, South San Francisco, CA, United States), CT values were normalized to β-actin, and relative expression was calculated by the Δ⁢ΔCT method. Assays-on-Demand Gene expression probes (Applied Biosystems) were as follows: AGRP Mm00435874_m1, POMC Mm01251072_g1, GPR101 Mm01296083_m1, PRDM12 Mm01324474_m1, and Prox1 Mm00435969_m1.

### MEA Recordings

Primary neurons were prepared as above with the entire hypothalamus being resuspended in 1 mL M5-5 media and plated on poly-L-lysine treated MEAs (60MEA200/30iR-Ti, Multichannel Systems, Reutlingen, Germany). Lids were made to cover the arrays from fluorinated ethylene-propylene (FEP) film (Scientific Commodities, INC. catalog # BB3090-1-24, Lake Havasu City, AZ, United States) attached to plastic cylinders with O-rings. MEAs were incubated at 37°C in 5% CO_2_ for 48 hrs before the M5-5 plating media was replaced with BrainPhys Neuronal Media (STEMCELL Technologies, Vancouver, BC, Canada) (Bardy et al., 2015). Every 10 ml of the media was supplemented with 200 μl NeuroCult SM1 neuronal supplement (STEMCELL Technologies), 100 μl N2 Supplement-A (STEMCELL Technologies), 20 ng/ml Recombinant Human Brain-Derived Neurotrophic Factor (STEMCELL Technologies), 20 ng/ml Recombinant Human Glial-Derived Neurotrophic Factor (STEMCELL Technologies), 1 mM dibutyryl cAMP (Santa Cruz Biotechnology), 200 nM ascorbic acid (STEMCELL Technologies), 1 μL/mL gentamycin and 10 μM ARA-C. A MEA2100-System (Multichannel Systems) with a headstage maintained at 37°C was used to acquire extracellular recordings from the cultures 10 days after they were plated on MEAs. Data was acquired from 60 electrode channels at a sampling frequency of 50 kHz per channel using MC_Rack and Multi-Channel Experimenter software (Multichannel Systems). Spontaneous field potentials were recorded during the addition of vehicle or drug. Media was removed from the MEAs for vehicle and drug dilutions. Baseline activity was recorded for several minutes in the presence of vehicle. While continuing to record, the following drugs were added to individual MEAs: leptin (10 nM in Tris; PeproTech Inc, Rocky Hill NJ; Cat. # 450-31 or R&D Systems, Minneapolis, MN, United States; Cat. # 498OB01M), MCH (1 μM in H_2_O; Tocris Bioscience, Minneapolis, MN, United States; Cat. # 3806100U), SAG (400 nM in DMSO). For priming experiments, cells were pretreated with 400 nM SAG 24 h before measuring activity in response to MCH. Action potential frequency was analyzed using a combination of MEA Tools within MEA Viewer (Bridges et al., 2018) and MATLAB scripting^1^. Spike data was extracted using MEA Tools, including spikes exceeding ±5X standard deviation of signal following a Butterworth filter. Data was then organized into a standardized matrix structure based on electrode layout, active electrodes, and timepoint before analysis. Electrodes were considered active when the baseline firing rate was greater than 0.5 Hz. Normalized firing rate was calculated by dividing the firing rate in the presence of drug by the baseline firing rate. Baseline firing rate was determined from the minute immediately preceding drug addition. Firing in the presence of drug was determined over a 60 s window during peak drug response (3 min following MCH addition, 4 min following leptin and SAG addition).

### Statistical Analysis

All statistical tests were performed using GraphPad Prism. All statistically significant observations are noted in the figures and specific tests used are within the legends.

## Data Availability

All datasets generated for this study are included in the manuscript and/or the supplementary files.

## Ethics Statement

All experimental procedures were approved by the Institutional Animal Care and Use Committee (IACUC) at the Indiana University – Purdue University Indianapolis.

## Author Contributions

RB, SE, and NB conceived and designed the experiments. RB, SE, PA, LW, AB, and NB performed the experiments. All authors analyzed the data. TC and NB contributed to reagents, materials, and analysis tools. RB, SE, JR, and NB wrote the manuscript.

## Conflict of Interest Statement

The authors declare that the research was conducted in the absence of any commercial or financial relationships that could be construed as a potential conflict of interest.

## Footnotes

1 https://github.com/Lswhiteh/MEA_Data_Analysis
